# Obstetrics: the Science and the Art

**Published:** 1857-02

**Authors:** 


*Obstetrics: the Science and the Art.* By Charles D. Meigs, M. D., Professor of Midwifery and the Diseases of Women and Children, in the Jefferson Medical College, at Philadelphia, etc., etc. Third edition revised. With one hundred and twenty-nine illustrations. Philadelphia: Blanchard & Lea.

The preface to this third edition is worthy of note. We give it entire:

"I have endeavored to fulfill the intention expressed in the last paragraph of the preface to the second edition of my work. I trust that my readers will find that I have made some amendments in the style, and that I have brought the subject up to the latest dates of real improvements in our art and science."

In previous notices of this and other works of Prof. Meigs, we have dwelt upon one great fault—a style now grand and finely descriptive, again tedious, verbose, inflated and trivial. The work on obstetrics was never so open to these objections as that on Diseases of Women and Children. It is now still less so. This is an *expurgated edition.*

As a teacher, Prof. Meigs has ever ranked high. His genius, enforced by unusual industry, has given him strong claims upon the confidence of the profession; while, at the same time, his peculiarities have made him the mark of decided criticism. At times acerbity of feeling has been generated by his sharp rejoinders to those who chose to freely use the critic's privilege of censure. His opinions on some points of pathology and practice are so at variance with the sense of other writers and teachers, that it is not probable that even his age, his high personal worth, or his acknowledged learning, will be sufficient to render them authoritative.

It is at once unnecessary and impossible for us to enter into any criticism, proper, upon the present volume. In the mechanism of labor, and the management of its immediate accidents, we can conceive of no better authority in the language, than Meigs. In the treatment of some puerperal diseases, the dissent which we have heretofore expressed is not weakened by the process of time, and further observations only serve to convince us of the erroneous character of his teachings. We allude especially to the pathology and treatment of puerperal fever. Prof. Meigs still asserts the non-contagious character of the one, and the efficacy of blood letting in the other. Both of these we regard as dangerous doctrines. To insist upon the non-contagion of child-bed fever, can be of no service to science commensurate with the heavy risks of human life it involves. Indeed we regard this doctrine as a far more serious error than any fault in treatment. The judgment of the practitioner usually prevents too strong reliance on the lancet at the bedside; but with a strong conviction that he cannot be the vehicle of this terrible poison, the physician may become a walking pestilence. Dr. Meigs should admit that there is at least a doubt, and that that doubt should suggest precaution, but with singular tenacity he will not admit even the possibility of danger.

For sale by PHINNEY & Co.

